# Ivermectin as a potential treatment for COVID-19?

**DOI:** 10.1371/journal.pntd.0009446

**Published:** 2021-06-01

**Authors:** Olivier Chosidow, Charlotte Bernigaud, Didier Guillemot, Bruno Giraudeau, Anne Lespine, Jean-Pierre Changeux, Hervé Bourhy, Marc Lecuit, Zahir Amoura

**Affiliations:** 1 Service de Dermatologie, Hôpital Henri Mondor, AP-HP, Université Paris-Est, Créteil, France; 2 Groupe de recherche Dynamyc, EA7380, Faculté de Santé de Créteil, École nationale vétérinaire d’Alfort, USC ANSES, Université Paris-Est Créteil, Créteil, France; 3 Université Paris-Saclay, UVSQ, Inserm, CESP, Anti-Infective Evasion and Pharmacoepidemiology Team, Montigny-Le-Bretonneux, France; 4 AP-HP, Paris-Saclay, Public Health, Medical Information, Clinical Research, Le Kremlin-Bicêtre, France; 5 Institut Pasteur, Epidemiology and Modelling of Antibiotic Evasion (EMAE), Paris, France; 6 UMR SPHERE Inserm U1246, Université de Tours, Université de Nantes, & INSERM CIC1415, CHRU de Tours, Tours, France; 7 INTHERES, Université de Toulouse, INRAE, ENVT, Toulouse, France; 8 Institut Pasteur CNRS UMR 3571 Department of Neuroscience and Collège de France, Paris, France; 9 Institut Pasteur, Lyssavirus epidemiology and neuropathology Unit, Paris, France; 10 Intitut Pasteur, Biology of Infection Unit, Inserm U1117, Paris, France; 11 Université de Paris, Service des Maladies Infectieuses et Tropicales, Hôpital Universitaire Necker-Enfants Malades, AP-HP, Institut Imagine, Paris, France; 12 Sorbonne Université, Inserm UMR-S 1135, Centre d’Immunologie et des Maladies Infectieuses (CIMI-Paris), AP-HP, Groupement hospitalier Pitié-Salpêtrière, service de médecine interne 2, Paris, France; George Washington University School of Medicine and Health Sciences, UNITED STATES

COVID-19 pandemic is affecting more than 164 million persons worldwide with 14% severe and 5% critical, with 3,4 millions of (reported) global deaths (https://coronavirus.jhu.edu). Management of severe/critical forms of COVID-19 has been greatly improved with the use of corticosteroids and anticoagulants based on Randomized Clinical Trials (RCTs), meta-analysis (MA) and observational studies [1,2]. Non-severe forms of COVID-19 are still uncontrolled, raising a double issue, *i*.*e*., the potential evolution to a severe life-threatening stage, and the transmission to the contacts of the infected index case. Although BNT162b2 vaccine might reduce SARS-CoV2 viral load after inoculation [3], to date, once a patient is infected, no-evidence-based treatment targeting those issues currently exists [4] and, therefore, any candidate drug should be properly evaluated, allowing robust conclusions for their use on a daily-basis at the population level. As it was initially suggested that oral ivermectin (IVM) might have an activity against SARS-CoV-2 *in vitro* [5], a RCT comparing IVM to placebo in mild COVID-19 was performed and the results were published in a recent issue of the JAMA [6]. The RCT was conducted from July 15 to December 21, 2020, by a single pediatric center in Cali, Colombia. Eligible SARS-CoV-2 RT-PCR or Ag positive adult patients were randomly assigned in a 1:1 ratio to receive either oral IVM 0.6% solution at a dosage of 300 μg/kg of body weight for 5 days or the same volume of placebo. The primary outcome was the time from randomization to complete resolution of symptoms within the 21-day follow-up period using an 8-category ordinal scale. In the statistical analysis subsection of the article, the authors acknowledged 2 RCT issues: *i)* they modified the primary end point to time from randomization to complete resolution of symptoms within the 21-day follow-up period and *ii)* a labeling error occurred between September 29 and October 15, 2020, resulting in an unblinded protocol during this time frame. Seventy-five patients were randomized during the unblinded period and were excluded from the primary analysis population. Of the 476 patients who underwent randomization, 238 were assigned to receive IVM and 238 placebo. The median age was 37 years (interquartile range (IQR), 29–48), and 231 (58%) were women). Time to resolution of symptoms in patients was not significantly different (median, 10 days vs 12 days; difference, −2 days [IQR, −4 to 2]. The proportion of patients who required escalation of care was smaller in the IVM group than in PBO but the difference was not significant, including in a post-hoc analysis (2% with ivermectin, 5% with placebo; difference, -3.05 [95% CI, -6.67 to 0.56]; OR, 0.38 [95% CI, 0.12 to 1.24]). The authors concluded that the usefulness of IVM for treatment of mild COVID-19 was not demonstrated.

We think that this RCT needs several clarifications that preclude drawing firm conclusions. First, we have a concern regarding the pharmaceutical/pharmacokinetics (PK) properties of the oral IVM solution used, and subsequently, its bioavailability: *i)* IVM is generally given orally as tablets in humans. An oral liquid formulation (for children), although claimed in 2020, is still expected [7]; indeed, solubilization of IVM has documented limitations due to its poor solubility in water (ranges between 0.04 and 0.005 mg/mL), the sole IVM oral solution that exists worldwide is for veterinary medicine, and FDA has recently reminded that this oral solution should not be given to humans (https://www.fda.gov/animal-veterinary/product-safety-information/fda-letter-stakeholders-do-not-use-ivermectin-intended-animals-treatment-covid-19-humans). Finally, the oral solution used for the RCT deserves further pharmaceutical details provided by the authors such as stability, excipients, and exact dose of IVM (as it was performed in a new formulation of solid self-emulsifying drug delivery system) [8]). *ii)* IVM was administered on an empty stomach although several PK data suggested its use with a meal in patients, to increase bioavailability and optimize potential efficacy [9,10]. Second, methods of the RCT also require comments: *i)* Placebo did not respect blindness in taste and smell in a subgroup of patients which represents about one third of the patients allocated to the IVM group. Even if only 1 patient per household was included, a bias cannot be excluded, notably because the outcome is not objective; *ii)* patients included had mild COVID-19 and, due to the natural history of the disease, the initially-defined primary end point, *i*.*e*. time from randomization to worsening by 2 points on an 8-category ordinal scale, was changed. Although we acknowledge that the authors fulfilled a reported process, *i*.*e*., change at the beginning of the trial with the agreement of the data safety monitoring board and a new sample size calculation -which was performed with more transparency than in other COVID-19 trials [11] -, such a change remains an issue. It may illustrate that, beyond this trial, outcomes used in many COVID-19 RCTs may not be well suited, maybe because these trials have been planned while one had poor knowledge on the disease at the time of onset of trials. Regarding outcomes, we may also wonder why effect on anosmia, an iconic and specific manifestation of COVID-19, easy to assess in clinical practice, was not measured (see below), although the variable was present at baseline. Last, the RCT was monocentric, limiting its external validity, and included a quite young population which is not expected to be at particular high risk of severe COVID-19 and is expected to recover rapidly, all points raising the issue of the relevance of the primary outcome (see above). Therefore, we consider that this RCT does not allow to draw a firm and definitive conclusion as regard to ivermectin in COVID-19, a conclusion also shared by the authors that acknowledge that “*larger trials may be needed to understand the effects of ivermectin on other clinically relevant outcomes*” (see below).

Indeed, several data suggest that IVM remains a good drug candidate for COVID-19. First, encouraging observational data exist [12]. Briefly, a 66-year-old woman (Resident 1) from a long-term care facility (LTCF-A), presenting profuse scabies and numerous comorbidities, was included in a scabies RCT, receiving either IVM 400 or 200 μg/kg (exact dose double-blinded, NCT02841215) on days 0, 7 and 14. Because of a scabies outbreak, other individuals of LTCF-A (68 residents and 52 staff members) received IVM standard dose (200 μg/kg). In parallel, 11 persons (7 residents and 4 staff members) presented confirmed/suspected COVID-19 (resident 1 had a positive SARS-Cov2 PCR); no hospitalizations and no deaths were noted versus a mean of 22.6% (95% CI, 16.3 to 28.9) acquired declared COVID-19 infections, with a lethality of 4.9% (95% CI, 3.2 to 6.5) in 45 matched (age, sex, LTCF fees and size) county-wide LTCFs (data from https://www.iledefrance.ars.sante.fr). Second, in the golden hamster model for COVID-19, a single subcutaneous injection of IVM at the dosage of 400 μg/kg reduced significantly the severity of clinical signs, including hyposmia/anosmia. This effect of IVM in SARS-CoV-2-inoculated hamsters correlated with a dampening of the inflammatory response in the lung (*i*.*e*., IL-6 in females) [13]. Interestingly, a small RCT (n = 12 for each group) comparing an early treatment with IVM (400 μg/kg single oral dose) vs placebo showed that IVM-treated patients recovered earlier from anosmia/hyposmia (76 vs 158 patient-days, p < 0.001) [14]. Third, a MA of 13 RCTs found that IVM reduced the risk of death compared with no IVM treatment (average risk ratio 0.32 (95% CI, 0.14 to 0.72; low to moderate-certainty evidence). IVM prophylaxis reduced COVID-19 infection by an average of 86% [15]. The potential mechanism of action of IVM is still unclear. The presumable anti-viral effect of IVM on monkey kidney (VeroE6) cells infected with SARS-CoV-2, shown previously (5,12), has not been confirmed on Calu-3 cells (Hakim Ahmed-Belkacem and Slim Fourati, personal communication). Results obtained in the golden hamster model suggest that IVM exerts an anti-inflammatory effect during experimental COVID-19. IVM is a positive allosteric effector of the alpha7 neuronal nicotinic acetylcholine receptor, and could therefore act via this receptor [16].

As drugs active in mild-to-moderate COVID-19 are still critically lacking, especially in patients at high-risk to develop a severe form, and the efficacy of vaccines may be jeopardized by emergences of SARS-CoV-2 escape mutants, testing IVM in well-designed multicentric RCTs is urgently needed. Health-authorities over the world might facilitate our request of including patients in such RCTS.

(https://www.covid19treatmentguidelines.nih.gov/antiviral-therapy/ivermectin/, https://www.ema.europa.eu/en/news/ema-advises-against-use-ivermectin-prevention-treatment-covid-19-outside-randomised-clinical-trials, https://ansm.sante.fr/actualites/lansm-publie-sa-decision-sur-la-demande-de-rtu-pour-livermectine-dans-la-prise-en-charge-de-la-maladie-covid-19, https://www.who.int/news-room/feature-stories/detail/who-advises-that-ivermectin-only-be-used-to-treat-covid-19-within-clinical-trials). Of note, moxidectin, another macrocyclic lactone with a longer half-life, FDA-approved for onchocerciasis, and currently investigated in scabies (NCT 03905265), should be ideally explored too.
